# Direct quality control of glycoengineered erythropoietin variants

**DOI:** 10.1038/s41467-018-05536-3

**Published:** 2018-08-21

**Authors:** Tomislav Čaval, Weihua Tian, Zhang Yang, Henrik Clausen, Albert J. R. Heck

**Affiliations:** 1 Biomolecular Mass Spectrometry and Proteomics, Bijvoet Center for Biomolecular Research and Utrecht Institute for Pharmaceutical Sciences, Science4Life, University of Utrecht, Padualaan 8, 3584 CH Utrecht, The Netherlands; 2 Netherlands Proteomics Center, Padualaan 8, 3584 CH Utrecht, The Netherlands; 3 Copenhagen Center for Glycomics, Department of Cellular and Molecular Medicine, Faculty of Health Sciences, University of Copenhagen, Blegdamsvej 3, DK-2200 Copenhagen N, Denmark

## Abstract

Recombinant production of glycoprotein therapeutics like erythropoietin (EPO) in mammalian CHO cells rely on the heterogeneous *N*-glycosylation capacity of the cell. Recently, approaches for engineering the glycosylation capacities of mammalian cells for custom designed glycoforms have been developed. With these opportunities there is an increasing need for fast, sensitive, and global analysis of the glycoproteoform landscape produced to evaluate homogeneity and consistency. Here we use high-resolution native mass spectrometry to measure the glycoproteoform profile of 24 glycoengineered variants of EPO. Based on the unique mass and intensity profiles of each variant, we classify them according to similarities in glycosylation profiles. The classification distinguishes EPO variants with varying levels of glycan branchingand sialylation, which are crucial parameters in biotherapeutic efficacy. We propose that our methods could be of great benefit in the characterization of other glycosylated biopharmaceuticals, ranging from the initial clonal selection to batch-to-batch controls, and the assessment of similarity between biosimilar/biobetter products.

## Abstract (web summary)

Several therapeutics are glycosylated proteins, yet the analysis of their specific glycosylation patterns remains challenging. Here the authors demonstrate an approach for the detailed characterization of glycosylated biopharmaceuticals applied to the determination of the glycoproteoform profile of glycoengineered variants of erythropoietin.

## Introduction

A large majority of clinically relevant protein-based biologics are glycosylated^1^. Glycosylation, specifically *N*-glycosylation, modulates the function of biologics in a variety of ways. Most notably, lack of a Fucose residue on *N*-glycans in the Fc domain of IgG molecules significantly increase antibody-dependent cell-mediated cytotoxicity^2^. Additionally, galactosylation and sialylation play an important role in complement-dependent cytotoxicity^3^ and anti-inflammatory activity^4^, respectively. Moreover, sialylation and increased branching of erythropoietin (EPO) *N*-glycans increases its serum half-life^5,6^, while EPO lacking sialylation exhibits neuroprotective role in vivo^7^. Thus, a well-defined view of the detailed glycosylation profile of biotherapeutics is essential.

**Fig. 1:**
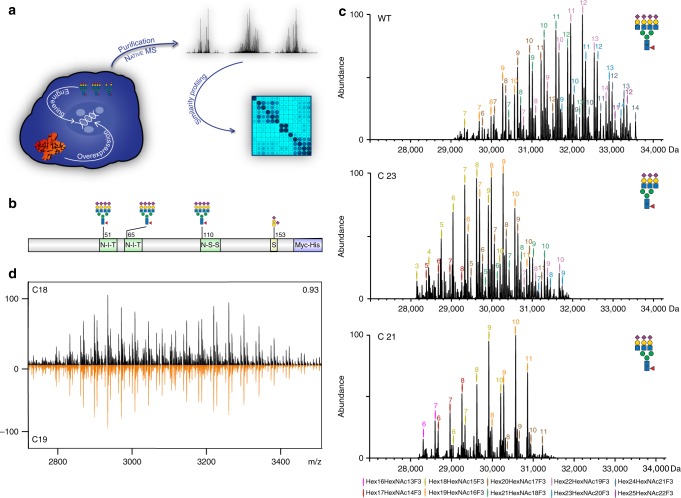
Generation and structural assessment of wild type and glyco-engineered EPO. **a** Depiction of the workflow used in this study. EPO is overexpressed in CHO cells whereby specific glyco-genes are manipulated to achieve the desired glycosylation, followed by EPO purification and native MS analysis. High-resolution native MS spectra of different clones are correlated, leading to a similarity matrix. **b** Depiction of EPO glycosylation sites. **c** Deconvoluted high-resolution native mass spectra of WT EPO (top) and two KO-based glyco-engineered clones: C23 (middle) and C21 (bottom). These two KO clones would supposedly delete β-4 and β-6branching of *N*-glycans as indicated by the pictograms. In these deconvoluted spectra the main glycoproteoforms are color coded, wherein each color corresponds to a unique Hex_x+3_HexNAc_x_F_3_ composition, where F denotes fucose, and the numbers placed above the annotated peaks indicate the number of sialic acid residues. **d** Comparison of native mass spectra of EPO from two biological replicate clones C18 (black) and C19 (orange).The correlation coefficient between these two spectra over all ion signals is 0.93, as depicted in the top-right. All other high-resolution native mass spectra of EPO, purified from each of the prepared 25 clones, are provided in Supplementary Fig 4

To ensure optimal efficacy and safety of biotherapeutics, human-like glycosylation with clearly defined glycoforms can be beneficial. For this reason, biologics are so far mostly expressed in Chinese hamster ovary (CHO) cells capable of human-like glycosylation^1^. However, CHO cells produce quite heterogeneous *N*-glycans on recombinant glycoproteins and also lack the capacity for producing the human-like α2,6-linked sialic acid glycoform. Thus, obtaining a homogenous biotherapeutic product with desirable glycan features remains a challenge. To address this need various glycoengineering approaches have been developed^8–13^. Attractive emerging strategies include the so-called GlycoDelete approach wherein complex *N*-glycans are reduced to trisaccharide stems^10,11^, and a second approach wherein combinatorial knock out (KO) and knock in (KI) of various glycosyltransferase enzymes in CHO are used to create a cell with custom designed glycosylation capacity to produce a more homogenous glycosylation profile^8,14^(Fig. 1a).

With biologics displaying such a complex glycosylation profile, an issue that needs to be addressed is the in-detail analytical assessment of the glycoproteoform heterogeneity. This is of special importance when a defined glycoprofile is required for a proper biological activity. In addition to that, looming patent expiry of almost all “blockbuster” biotherapeutics, will open avenues for opportunities in creation of biosimilars and/or bio-better biologics, that need to be structurally and functionally evaluated in comparison to their corresponding originals^1,15,16^.

A direct assessment of the structural similarity may come from the high-resolution mass measurement of the glycoprotein of interest in its intact form. However, even for IgGs, that express typically a relatively simple glycosylation profile, measuring under standard reducing MS conditions will not provide a complete picture of glycoproteoform heterogeneity^13^. This issue was partially addressed with the introduction of an Orbitrap mass analyzer with extended mass range (Orbitrap EMR)^17^. The Orbitrap EMR enabled high-resolution mass spectrometry (MS) measurements of intact IgG glycoproteoforms under non-denaturing/native conditions^18^. What makes native MS attractive for IgG analysis is the simplicity of the resulting spectra and efficient analysis, which can typically be done under an hour^19^. Furthermore, when benchmarked against more standard glycomics approach, glycoproteoform profiling of intact IgGs by native MS performed qualitatively as well as quantitatively very well^20^. However, the complexities with glycoproteins having multiple glycosites and both N and O-linked glycans have so far not been overcome, although great strides towards this goal have been achieved^21^. Here, we approach EPO^22^, which is one of the most used therapeutic biologics today and has a complex and heterogeneous glycoprotein with three *N*-glycans and one O-glycan^23^. When compared to the relatively simple native MS spectra for therapeutic IgGs that typically display only a dozen of different glycoforms, the EPO native MS spectra exhibit hundreds of different glycoproteoforms^22^ arising from the heterogeneity on its three *N*- and one O-glycosylation sites. This makes compositional analysis of such spectra much more challenging.

Here we explore and demonstrate the applicability of native MS for the structural characterization of engineered variants of EPO with diverse patterns of *N*-glycans. We used a panel of glycoengineered CHO cells to express EPO with different glycosylation features and degrees of heterogeneity to dissect and validate the native MS spectra. For this purpose, we targeted 24 differentially glycoengineered EPOs, all expressed in CHO cells. The observed glycosylation profiles of the glycoengineered EPOs reveal the co-occurrence of hundreds of variants, which show a stepwise decrease in heterogeneity as we progress through our glycoengineering scheme—from heterogeneous, tetra-antennary, polyLacNAc elongated *N*-glycans all the way to homogenous, bi-antennary, non-elongated, and non-sialylated *N*-glycans. We demonstrate the potential of native MS, and first fully assign the complex glycosylation profile of wild-type (WT) EPO. For illustrative purposes, we next focused on the in-depth analysis of two engineered EPO variants, i.e. the *mgat5* KO and the stacked KO of *mgat4A* and *mgat4B*. Although both these clones express tri-antennary dominated *N*-glycan profiles, albeit with different branch points, our data reveal clear differences, whereby the *mgat4A/4B* KO clone exhibits a much more complicated glycoprofile, originating from a substantial increase of polyLacNAc extensions, revealing that native MS is capable of distinguishing close isomeric configurations of triantennary *N*-glycans.

Finally, focusing on all analyzed 24 EPO variants we introduce a classification scheme, based on the acquired native MS spectral fingerprints. We demonstrate that just by comparing native mass spectra a product similarity matrix can be created. Using hierarchical clustering we can directly differentiate glycoengineered EPO clones, and extract what the gross structural differences are. In this matrix, we distinguish discrete clusters of 2-, 3-, 4-antennary modified EPOs, and clusters exhibiting high and low sialylated EPO variants. Our approach for classification, and biosimilarity scoring, is generic and could benefit the characterization of other glycosylated proteins, clonal selection in cell line development, and be used for batch-to-batch quality control (QC) of the glycoproteoform profiles and assessment of structural aspects linked to biosimilarity.

## Results

### Characterization of WT EPO glycoproteoform profile

Most glycoengineered EPO clones in our study were designed to reduce glycoproteoform heterogeneity compared to the non-glycoengineered CHO-cell produced wild type (WT) EPO. Therefore, in principle the WT EPO sample should provide the most complex glycosylation profile. Thus, we first attempted to analyze and assign as many structural variants in WT EPO, based on the acquired high-resolution native MS data. The acquired native MS data on WT EPO were in line with previously published data on commercially available recombinant EPO variants^22^. To illustrate the description of our EPO assignments we display in Fig. 1b a graphic representation of one of the most abundant WT EPO glycoforms. Depicted are 3 tetra-antennary *N*-glycans and one O-glycan (T structure, Galβ1-3GalNAc) that together result in a total glycan composition of Hex_22_HexNAc_19_Fuc_3_ (using standard nomenclature^24^) and 14 sialic acids (sia)—which is the maximum amount of sia moieties a single EPO molecule can contain. Any further increase in HexHexNAc content stems from the elongation of one of the antennae in a process termed poly-*N*-acetyllactosamine (LacNAc) elongation. In the “zero-charge” deconvoluted native mass spectrum of WT EPO (Fig. 1c top) we see that the Mw distribution of EPO glycoproteoforms ranges from 29,000 up to almost 34,000 Da. This 5000 Da spread is caused by differential glycosylation where the main glycan compositions correspond to a generic composition of Hex_x+3_HexNAc_x_Fuc_3_Sia_y_, wherein 15 < x < 22, and 7 < y < 14. The most abundant glycoproteoform contains Hex_22_HexNAc_19_Fuc_3_, as depicted in Fig. 1b, albeit containing 12 instead of 14 sia moieties. Additionally, we observed other glycoproteoforms on WT EPO that are further modified by polyLacNAc elongation (Hex_23-25_HexNAc_20-22_Fuc_3_Sia_7-14_), and/or sequential losses of 1 to 4 of *N*-glycan branches (Hex_18-21_HexNAc_15-18_Fuc_3_Sia_7-13_), resulting in a mixture of bi-, tri-, and tetra-antennary *N*-glycans. Evidently, there is always a possibility of multiple isobaric species present within the same narrow mass window. For instance, the Hex_22_HexNAc_19_Fuc_3_ composition could correspond to 3 tetra-antennary *N*-glycans and an O-glycan, but the same composition could also represent 1 tetra-antennary *N*-glycan, 2 polyLacNAc elongated tri-antennary *N*-glycans and no O-glycan. To tackle this issue and probe the possible presence of isobaric species within each signal we inspected the native MS spectrum of the *B3gnt2* KO that is responsible for polyLacNAc elongation in CHO cells^8^ (Supplementary Fig. 1b). In the native MS spectrum of EPO extracted from clone C16 (*B3gnt2* KO), we were surprised to see that each of the Hex_18-21_HexNAc_15-18_Fuc_3_ glycoproteoforms remained almost identical to that of the WT EPO indicating that each of these glycoproteoforms stems predominantly from the loss of one of the branches on the *N*-glycans. On the other hand, we observed the complete disappearance of glycoproteoform compositions containing Hex_23-25_HexNAc_20-22_Fuc_3_ when compared to WT EPO, indicating that these compositions in WT EPO are indeed polyLacNAc elongated.

From the frequently observed Δm-shift of 291 Da it is apparent that a major factor in EPO glycoproteoform diversity is differential sialylation. On average, in EPO each Hex_x+3_HexNAc_x_Fuc_3_ composition exhibits 6 different states of sialylation, with compositions carrying 11 and 12 sialic acid moieties representing the most abundant variants. To reduce the heterogeneity, we treated WT EPO with a broad specificity sialidase enzyme and reanalyzed that sample by native MS (Supplementary Fig. 1c). Inspection of this, much less dense, mass spectrum facilitated the assignment of 12 distinct Hex_x+3_HexNAc_x_Fuc_3_ (*x* = 14–25) glycan compositions, 6 additional glycoproteoforms with additional HexNAc moieties, and 6 glycoproteoforms with Δ*m* = 80 Da adducts. Although we cannot distinguish sulfation and phosphorylation directly from our native mass spectra (Δ*m* = 0,00951 Da), based on the biosynthetic pathways of *N*-glycans and previous studies^25,26^ the 80 Da adducts most likely originate from sulfation. Evidently, all these structural variants are also present in the non-sialidase treated WT EPO sample. However, due to heterogeneous sialylation present in the untreated sample their annotation becomes more ambiguous and requires additional levels of analysis, such as we do here by pre-treating WT EPO with sialidase. Finally, based on the EPO data from the *B3gnt2* KO clone and the sialidase treatment, we could extract that the maximum sialylation of each Hex_x+3_Hex_x_NAcFuc_3_ glycan is indicative of its *N*-glycan branching. For instance, Hex_22_Hex_19_NAcFuc_3_ has a maximum sialic acid content of 14 indicating the presence of three tetra-antennary *N*-glycans. Upon the loss on one HexHexNAc, which translates into one tri-antennary and two tetra-antennary *N*-glycans the maximum number of sialic acid should be 13, and indeed the composition of Hex_21_HexNAc_18_NAcFuc_3_ does contain a maximum of 13 sialic acid acid moieties. In agreement, if we inspect the Hex_20_HexNAc_17_NAcFuc_3_ peak, we observe its glycan distribution ends at 12 sialic acid moieties indicating the presence of yet another tri-antennary *N*-glycan.

### Glycoproteoform profiles of individual glycoengineered EPO’s

Following the detailed analysis and assignments of structural features observed in WT EPO, we next focused on the high-resolution native MS data of all variants. To describe how individual KOs and KIs affect the overall EPO glycoproteome profile, we describe in detail two examples, namely C21 EPO, which represents a KO for the β1,6-*N*-acetyl-glucosaminyltransferase (*mgat5*) involved in the β6-branching of *N*-glycans, and C23 EPO, which represents a stacked KO of the β1,4-*N*-acetylglucosaminyltransferase isozymes A and B (*mgat4A/4B*) that control the β4-branching of *N*-glycans. Because both KO clones express triantennary glycans^8^, we hypothesized that the resulting EPO glycoproteoform profiles would look similar. The deconvoluted native MS spectra are displayed in Fig. 1c middle and bottom spectra, for the *mgat4A/4B* KO and *mgat5* KO, respectively. Comparing these spectra with each other, but also with the spectrum obtained for WT EPO (Fig. 1c top), it is directly evident that the KO of *mgat5* results in a more simplified spectrum for EPO when compared to *mgat4A/4B* and WT EPO. Through spectral inspection and annotation, using the same reasoning as presented above for WT EPO, we conclude that the more simplified glycoproteome profile for *mgat5* KO originates from a substantial decrease in polyLacNAc content (Supplementary Fig. 2). This can be extracted from the low abundance of the Hex_20_HexNAc_17_Fuc_3_ variants, which indicates the presence of at least one polyLacNAc repeat, and complete absence of Hex_21-22_HexNAc_18-19_Fuc_3_ compositions that are present in both *mgat4A/4B* KO and WT EPO. From these observations, we could confirm that the main acceptor site for polyLacNAc elongation on *N*-glycans is specifically the β6-branch and not the β4-branch.

Additionally, we detected a substantially higher diversity and degree of sialylation in *mgat4A/4B* KO EPO, when compared to *mgat5* KO. For instance, the two most abundant glycan composition in both sets of KOs; Hex_19-18_HexNAc_16-15_Fuc_3_, harbor anywhere between 3–11 sialic acid moieties in *mgat4A/4B* KO, while it contains predominantly just 6–11 sialic acid moieties in *mgat5* KO. This difference may be due to either incomplete sialylation or the presence of multiple isobaric varieties exhibiting the same mass. Considering the above-described analysis of WT EPO and *B3gnt2* KO, we argue that these notable differences are mainly due to the incomplete sialylation occurring in *mgat4A/4BKO*, which is also found with the released N-glycan profiling^8^.

### Direct structural assessment of glycoengineered EPO variants

**Fig. 2:**
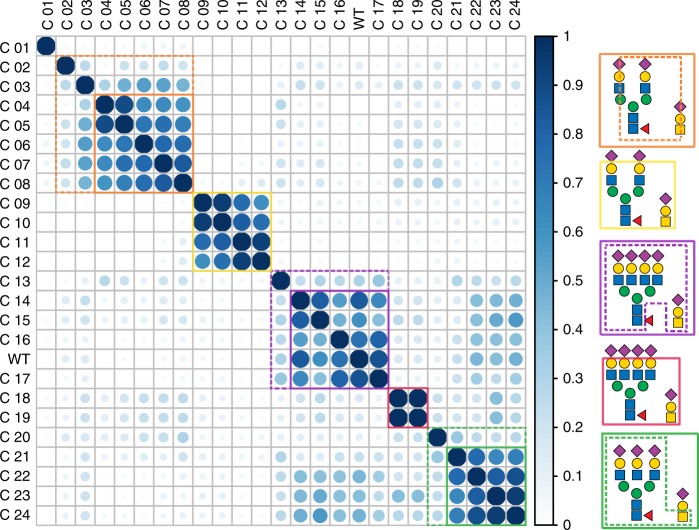
Clustering of glycoengineered EPO clones based on the correlation between their native MS spectra. In Supplementary Table 1 an overview is given of all 25 clones used, annotating which enzyme(s) was/were knocked-out/knocked-in. Color and size of the circles indicate the similarity between clones where 1 (full circle, dark blue) represents nearly identical EPO glycoproteoform profiles. Clusters boxed by full lines represent clones that share high similarity in their profiles in terms of *N*-glycan branching, sialylation and O-glycosylation capacity. Cluster expansions within dashed lines represent clones that display similarity as well as subtle differences in terms of *N*-glycan sialylation or O-glycosylation capacity. Orange, Green, and Purple boxes represent EPO clones expressing primarily bi-, tri-, or tetra-antennary *N*-glycans, respectively. Wild Type EPO is denoted as WT

Detailed analysis of all glycoproteoforms distinguishable in the high-resolution native mass spectra is laborious and still dependent on manual inspection. However, a significant benefit of native mass spectra is that an integral picture about glycoproteoform complexity is obtained within a single spectrum. If we consider the native mass spectra as simple barcodes for EPO produced by each clone, we could significantly accelerate data analysis of novel clones by just finding the most similar clone in a larger set of annotated spectra. Moreover, if one uses the same clone in a batch mode and wants to ensure the product consistency, the native MS spectra can provide a similarity score used for QC^22^. To test the reproducibility of the native MS spectral readout we measured and compared EPO produced by the C18 and C19 clones (Fig. 1d), being biological replicates, albeit created and characterized 20 months apart. We observed that EPO purified from these two clones displays an almost identical glycoproteoform pattern, reflected by a very high similarity score of 0.93. To illustrate the potential of using the spectral barcodes to classify glycoengineered EPO variants, we analyzed 25 EPO variants and determined their spectral correlations. Using unsupervised hierarchical clustering we constructed a matrix of all EPO variants, as shown in Fig. 2. The specific enzyme KO used in each clone is listed in Supplementary Table 1 and the full-unprocessed native MS spectra of all EPO variants are provided in Supplementary Fig. 4. The clustering led to 5 distinct clusters, and one extra single clone, which seemingly does not belong to any of these 5 clusters. The first cluster (solid orange box, C04-08) represents predominantly biantennary sialylated EPO variants. A slightly more extended cluster (dashed orange lines) includes additionally C03, which harbors a mixture of mono- and bi-antennary glycans, and C02 that has an alike N-glycan profile as C04-08 but carries truncated O-glycans in the form of STn antigens.

In the second cluster (yellow box), we find EPO from 4 clones; C09-C12, C09 and C10 are biological replicates that express biantennary *N*-glycans with reduced sialylation. Interestingly, they show an increase in polyLacNAc containing glycoproteoforms when compared with the C08 clone, which exhibits more sialylation, indicating that sialylation may limit the extent of polyLacNAc elongation. C11 and C12 represent a set of biological replicates that in addition to diminished sialylation carry an additional KO eliminating polyLacNAc, resulting in an even more uniform glycoproteform profile. Taken all together this further demonstrates both the reproducibility of the method as well as sensitivity to detect the presence of polyLacNAc species.

A third clear cluster (purple box) reveals that wild type EPO and EPO from clone C17 share almost identical glycoproteoform profiles, which was expected as the *B4galt4* KO used in the C17 clone does not have compromised galactosylation capacity for *N*-glycans^8^. EPO from C16, which also falls in this cluster, exhibits a slight decrease in similarity, largely due to a decrease in polyLacNAc containing glycoproteoforms. A direct comparison of the polyLacNAc and sialic acid content of EPO from the two clones C15 and C16, which display the highest level of dissimilarity in this cluster, is shown in Supplementary Fig. 3. Striking differences are found in the amount of sialylation and polyLacNAc elongation with EPO from C16 being more sialylated, while EPO from C15 being more polyLacNAc elongated. EPO from the other two clones in the same cluster (C14 and C15), are both KOs of enzymes that do not influence glycosylation in CHO cells. This means that we can almost treat EPO WT, C14-15 and C17 as biological replicates. Indeed, the subtle differences between these 4 clones are primarily in their sialylation status. EPO WT and C 17 contain highly sialylated *N*-glycans (on average 12 sialic acid moieties per glycoproteoform), on the other hand EPO from C14 and C15 exhibit a substantially lower sialylation with on average 8 sialic acid moieties per glycoproteoform. We hypothesize that a decrease in sialylation of these two clones is caused by variations during clonal development and expansion. Furthermore, the C13 clone, which can be found in the same, albeit extended cluster (dashed purple lines), is a KO blocking only core fucosylation. When compared to WT EPO its glycoproteoform profile is characterized by a −438 Da mass shift of each observed signal, corresponding to the loss of 3 fucose moieties, which further supports our annotations of WT EPO in Fig. 1c. Another cluster (red box) contains EPO from the above described C18 and its biological replicate C19 that contain tetra-antennary *N*-glycans with diminished sialylation, and as such is not expected to show similarity to the purple cluster containing highly sialylated tetra-antennary *N*-glycans. This data reinforces the strength of the method for quality assessment of EPO produced in different clones and batches.

A fifth cluster (green box) includes EPO from C21 and C23, which were analyzed in depth, as discussed above (Fig. 1c bottom and middle), C24, and surprisingly C22. Based on the supposed combinatorial KOs of the *mgat4A/4B/5*, *st3gal4/6*, and *cosmc/C1GALT1* enzymes, C22 was expected to produce EPO with non-sialylated biantennary *N*-glycans, and exhibit truncated O-glycosylation. However, as clearly deduced from the clustering analysis, the glycoproteoform profile was found to be very similar to that of C24 EPO, indicating that it was more likely a clone with KO of mgat4B (see Supplementary Fig. 5). Thus, our initial data hinted at a possible mix up of samples during some stage of sample purification and handling. Indeed, we could confirm this by subsequent repeated genotyping of C22 demonstrating that the EPO sample indeed was derived from a *mgat4B* KO CHO clone. Related to this cluster (dashed green lines) EPO from C20 shares similarity with the other triantennary clones. Again, this can be rationalized, as C20 should express triantennary *N*-glycans, with most differences originating from truncation of O-glycans.

Finally, in our dataset there is a single clone that does not take part into any of these 5 clusters; C01. The C01 clone is an *mgat1* KO and thus expresses only core *N*-glycans with up to 5 mannose moieties and limited amounts of core fucosylation, and as such is not expected to share similarity with any of the other clones which all express complex type *N*-glycans.

### KO of st3gal4 and st3gal6 does not abolish sialylation

**Fig. 3:**
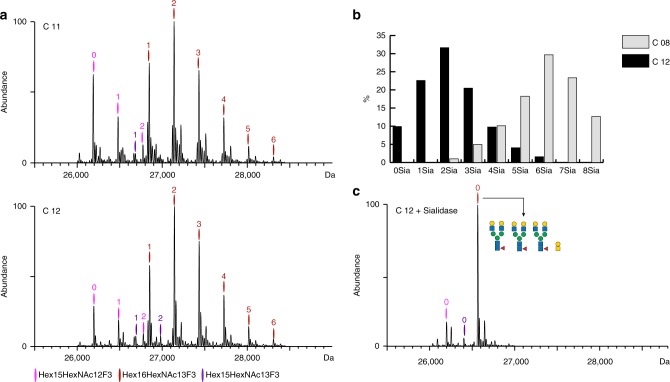
Biological reproducibility and sialylation status of EPO clones expressing bi-antennary *N*-glycans. **a** Deconvoluted mass spectra of EPO from two biological replicate clones, C11 (top) and C12 (bottom) exhibiting variable degree of O-glycosite occupancy (Hex_15_HexNAc_12_F_3_ glycoproteoform), **b** Comparison of sialylation on EPO purified from clone C08 (*mgat4A/4B/5* KO) and C12, whereby the latter has an additional *st3gal4/6* KO. **c** Deconvoluted native mass spectrum of sialidase treated EPO purified from the C12 clone. The depicted glycan composition corresponds to the total glycan content of the most abundant mass peak. Main glycoproteoforms are color coded, wherein each color corresponds to a unique Hex_x+3_HexNAc_x_F_3_ composition, and the numbers above the annotated peaks indicate the number of sialic acid residues

Although CHO cells produce human-like glycosylation there is one major difference. The sialic acids, being the outermost sugar moieties observed on *N*-glycans are attached via α2-3 linkages in CHO as opposed to human cells that in addition have attached α2-6 linkages. Although the direct consequences of this key difference in linkage on drug performance are not yet known, it is desirable to produce EPO with all possible human structural features. We previously reported that the combined KO of *st3gal4/6* essentially abolished sialylation of *N*-glycans^8^. This was based on MALDI-TOF of released permethylated glycans, however, when we here measured EPO by native MS we were able to detect low levels of glycoproteoforms with up to 6 sia moieties (Fig. 3a top and bottom). Interestingly, site-specific analysis of these EPO samples by LC-MS/MS also failed to detect the presence of sialic acids (not shown). Since only 2 out of these 6 sia moieties could originate from the O-glycan on EPO (Supplementary Fig. 6), the residual 4 sia moieties must originate from *N*-glycans. Although double KO of *st3gal4/6* clearly decreased the sialylation levels markedly (Fig. 3b), we could show that it did not completely eliminate the presence of *N*-glycan sialylation. This indicates that additional *st3gal’s* need to be knocked out to obtain EPO devoid of sialic acid on *N*-glycans, and *st3gal3* is expressed in CHO and triple *st3gal3/4/6* KO clones have been generated^8^. These findings are further supported by the observation of up to 4 sia moieties in EPO from the C18 clone, shown in in Supplementary Fig. 7, that is also a *st3gal4/6 KO*, but contains tetra-antennary *N*-glycans.

We noticed a −18 Da peak trailing each of the peaks corresponding to Hex_16_HexNAc_13_Fuc_3_Sia_1-4_ compositions. This peak completely disappeared upon sialidase treatment (Fig. 3c), indicating that the −18 Da peak is caused by a dehydration event on the sialic acid moiety. Due to the presence of dehydrated peaks in both biological replicates, corroborated by a recent study^27^ describing the presence of dehydrated sialic acids in commercially available bio-therapeutic EPOs we are inclined to believe that this is not an artefact caused by the ESI process, but rather a biologically occurring phenomena. Finally, we also observed a significant bias of standard bottom up approach for glycopeptide quantitation which showed that about 60% of the O-glycosite is occupied in these samples^8^. Previous studies have estimated 70–80% occupancy^28^, but our native MS data (Supplementary Fig. 6) indicates that the occupancy is rather near complete (90%). This further solidifies the benefit of our approach for obtaining a true glycoproteoform profiles.

## Discussion

We present a comprehensive analytic flow of the complex glycoprotein EPO using a consecutive series of glycoengineered EPO variants with and without specific glycan features, and we demonstrate that native MS offers an excellent tool to quantitate glycoproteoforms and discern even minor changes in glycan structures. However, it is important to note that this type of data can be acquired only on an orbitrap EMR instruments. We hope that continuous advancements in instrument development will enable other platforms to generate such data as well.

When compared with our previous study of glycoproteins^22^, which combined middle-down and intact native MS analysis our work represents a significant advancement in two ways. Firstly, by eliminating the need for middle-down analysis we no longer require time-consuming sample preparation and glycopeptide enrichment steps which shortened the analysis time from weeks to a single day. Secondly, and more importantly, MS analysis of glycoproteins or glycopeptides usually allows only for compositional profiling of glycans attached to the backbone, i.e only the total mass of the glycan attached can be deduced, but information about underlying structure remains elusive. However in this study by combining the glyoengineering efforts with native MS we were able to move beyond compositional profiling of glycans and discern glycan brancing vs. polyLacNAc elongation isomerism. We forsee that with this methodology it will ultimately become feasible to completely deconstruct a given glycosylation profile of other glycoproteins.Thus, native MS is an excellent tool for screening consistency in glycoprotein products as well as providing a much-needed rapid profiling tool for selection of cell clones during cell line developments for recombinant therapeutics. Moreover, it will become increasingly more important to study divergence between biosimilars and originators as well as different biosimilars of the same originator product^29^ that arises from the modifications in the production processes during the product lifecycle^30^ where native MS would prove especially advantageous.

## Methods

### EPO samples

Recombinant human EPO was produced in a panel of gene engineered CHO cell lines as previously described^8^. CHO media, supplements and other reagents were obtained from Sigma– Aldrich unless otherwise specified. CHO cells were maintained as suspension cultures in CHO CD Fusion serum-free media, supplemented with 4mM l-glutamine in 50 mL TPP TubeSpin® Bioreactors with 200 rpm or 100 ml in Corning 500 ml culture bottle with 130 rpm (Infors, USA) at 36.5 °C and 5% CO_2_ in air. Gene engineering was performed in CHOZN GS−/− cells (Sigma-Aldrich) or in CHO-K1 (ATCC) by ZFN mediated KO (*mgat1/3/4* *A/4B/5, st3gal4/6*, *B3gnt1/2*, *B4galt3/4*, *cosmc*) and by site-directed ZFN mediated KI using the ObLiGaRe strategy, as previously described^8^. Two engineering events were performed with TALENs (*mgat2*) or CRISPR/Cas9 (*fut8*). Briefly, CHO cells were transfected with 2 μg of each ZFNs or TALENs tagged with GFP/Crimson, and for KI with additional 5 μg donor plasmid, using Amaxa Nucleofector 2B (Lonza, Amaxa kit V, U24). After transfection cells were enriched by FACS and the pool after 1–2 weeks was further single cell sorted to 96 well plates on a BD FACSARIA III cell sorter. Clones were screened by Indel Detection by Amplicon Analysis (IDAA) and indels further verified by Sanger sequencing^31^. Targeted gene KI clones were screened by immunocytology with monoclonal antibodies to the introduced enzymes and further verified by junction PCR. Expression constructs containing the entire coding sequence of human EPO cloned into pcDNA3.1/myc-His (C-terminal tags) respectively, were synthesized by Genewiz, USA, and stable expression of EPO was established in the gene engineered clones by transfection of the plasmid DNA and Zeocin selection. EPO is a 193 amino acid protein with *N*-glycans at Asn51, Asn65, and Asn110, and O-glycan at Ser153. His-tagged recombinant human EPO was purified by nickel affinity purification (Invitrogen, US). Media was mixed 3:1 (v/v) in 4x binding buffer (200 mM Tris, pH 8.0, 1.2 M NaCl) and applied to 0.3 ml packed NiNTA agarose (Invitrogen), pre-equilibrated in binding buffer (50 mM Tris, pH 8.0, 300 mM NaCl). The column was washed with binding buffer and then bound protein was eluted with binding buffer with additional 250 mM imidazole. Fractions containing EPO were determined by SDS-PAGE and further purified on a reverse-phase HPLC purification with a Jupiter C4 column (5 µm, 300 Å, column 250 × 4.6 mm) (Phenomenex), using 0.1% trifluoroacetic acid and a gradient of 10–100% acetonitrile. Purity of proteins was evaluated by Coomassie SDS-PAGE and proteins were quantified by BCA Protein Assay Kit (Thermo Scientific, Rockford, US).

### Sample preparation for native MS analysis

20 μg of each EPO sample was buffer exchanged into 150 mM aqueous ammonium acetate (pH 7.5) by ultrafiltration (vivaspin500, Sartorius Stedim Biotech, Germany) with a 10 kDa cut-off filter. Concentration was adjusted to 5 μM and 4 μL was used for native MS analysis. Part of the EPO samples were treated with 0.02 U of sialidase (Roche, IN, USA) or four units of PNGase F (Roche, IN, USA) and incubated at room temperature overnight. Following the incubation EPO samples were buffer exchanged once more into 150 mM aqueous ammonium acetate (pH 7.5) before native MS analysis.

### Native MS analysis

Samples were analyzed on a modified Exactive Plus Orbitrap instrument with extended mass range (EMR) (Thermo Fisher Scientific, Bremen) using a standard *m*/*z* range of 500–10,000. The voltage offsets on transport multi-poles and ion lenses were manually tuned to achieve optimal transmission of protein ions at elevated *m/z*. Nitrogen was used in the HCD cell at a gas pressure of 6 × 10^−10^ bar. MS parameters used: spray voltage 1.2–1.3 V, source fragmentation and collision energy were varied from 5–30 to achieve optimal desolvation, and resolution (at *m/z* 200) of 17,500. The instrument was mass calibrated in 500–5000 m/z range using CsI clusters as described previously^19^.

### Data analysis

Average masses of Hexose (Hex, 162.1424), *N*-acetylhexosamine (HexNAc, 203.1950), fucose (F,146.1430) and sialic acid (291.2579) were used for PTM annotations. Based on the known backbone mass of EPO and biosynthetic pathway of *N*-glycosylation a matrix of possible glycoproteoforms was constructed. This matrix was then used to annotate raw spectra of EPOs and the annotation was considered correct if the corresponding proteoform could be found in at least 3 consecutive charge states. Raw spectra were then further deconvoluted to zero-charge by Intact Mass software^32^ (Protein Metrics, CA, USA) using default settings, except for the mass range, which was adjusted based on the minimum and maximum mass proteoforms identified directly from the raw spectra to minimize the presence of artificial peaks. For quantitation of proteoforms, intensities were extracted directly from raw spectra and were summed across the identified charge states.

### Clustering analysis

Raw spectra were pre-processed by binning the data points for each peak into defined *m*/*z* range. Bin size of 0.1 *m*/*z* was chosen as an optimal bin size that maximizes the differentiation of partially overlapping peaks between different raw spectra. Hierarchical clustering (complete linkage algorithm) using Pearson correlation was used to identify spectra with similar glycosylation features. Corresponding similarity matrix was produced using the freely available R package corrplot (version 0.84).

### Data availability

The data and the raw files that support the findings of this study are available on request from the corresponding author (A.J.R.H.). All cell lines are available on request under a standard MTA with University of Copenhagen for academic research purposes.

## Electronic supplementary material

Supplementary Information
