# Supplementary material for: Direct quality control of glycoengineered erythropoietin variants
Source: Nat Commun. 2018 Aug 21;9:3342. doi: 10.1038/s41467-018-05536-3 (PMC6104044; doi:10.1038/s41467-018-05536-3)
Supplement: Supplementary file 1 — Supplementary Information [file 41467_2018_5536_MOESM1_ESM.pdf]

Direct Quality Control of Glycoengineered Erythropoietin Variants

Tomislav Čaval, Weihua Tian, Zhang Yang, Henrik Clausen and Albert J.R. Heck

SUPPLEMENTARY INFORMATION

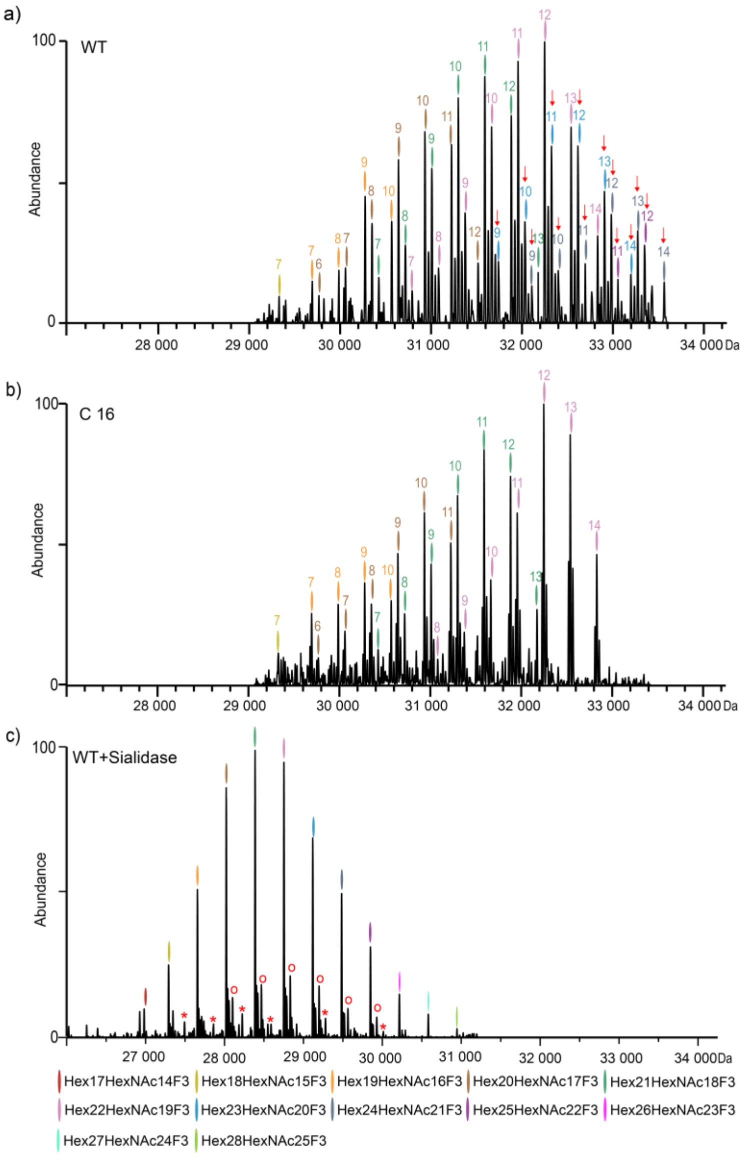

Supplementary Figure 1. Probing the presence of EPO polyLacNAc elongation in glyco-engineered EPO**.** Deconvoluted native mass spectra of **a.** WT EPO (top), **b.** EPO extracted from the C16 clone (middle), which is a KO for polyLacNAc elongation (*B3gnt2* KO) and **c.** sialidase treated WT EPO (bottom). The main glycoproteoforms are color coded, wherein each color corresponds to a unique $Hex_{x+3}HexNAc_xF_3$ composition, and the numbers above the annotated peaks indicate the number of sialic acid residues. Compositions indicated by red arrows in the top spectrum vanish upon polyLacNAc KO, as seen in the middle spectrum. Red stars and circles in the bottom spectrum indicate additions of HexNAc and 80Da moieties, respectively.

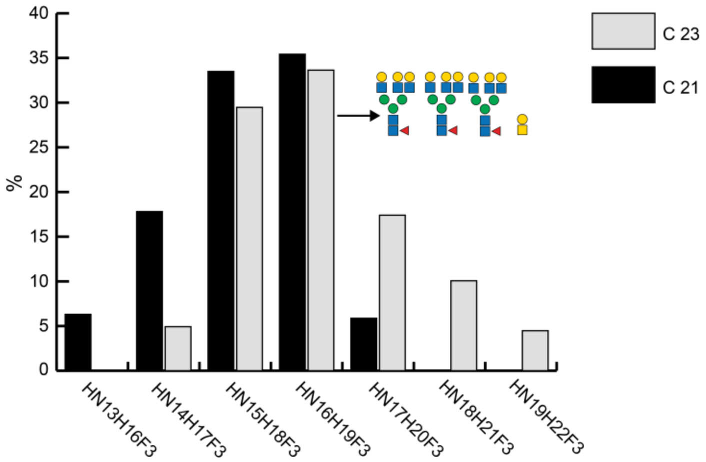

Supplementary Figure 2. Comparison of polyLAcNAc content of EPO extracted from the C21 (*mgat5* KO) and C23 (*mgat4A/4B* KO) clones. Drawn is the composition for glycoproteoforms with the generic $Hex_{19}HexNAc_{16}F_3$ composition, all abundant light grey compositions to the right are polyLacNAc elongated.

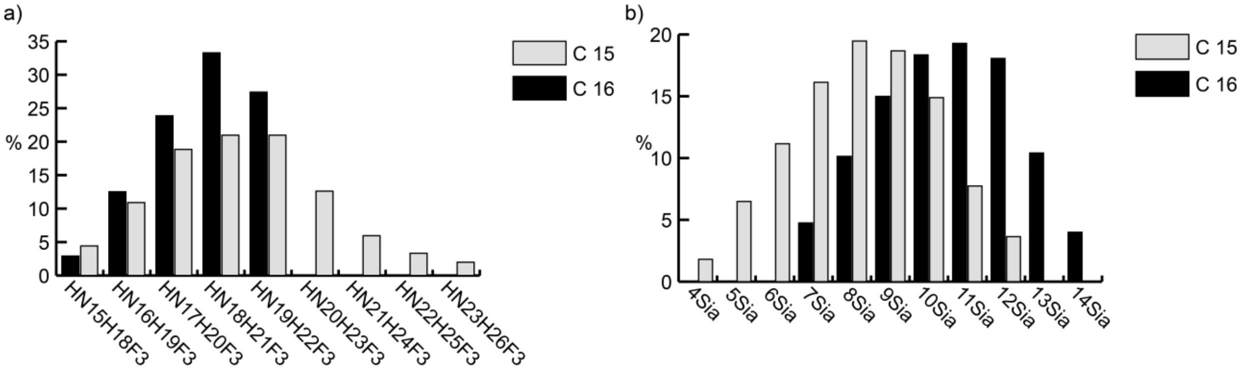

Supplementary Figure 3. PolyLacNAc and sialylation status characterization of *B3gnt1* KO and *B3gnt2* KO**. a** Distribution of polyLacNAc extensions on EPO purified from the clones C15 (*B3gnt1* KO) and C16 (*B3gnt2* KO), HN19H22F3 corresponds to the maximal composition with zero polyLAcNAc elongations. **b.** Distribution of sialic acid content on EPO purified from the clones C15 and C16.

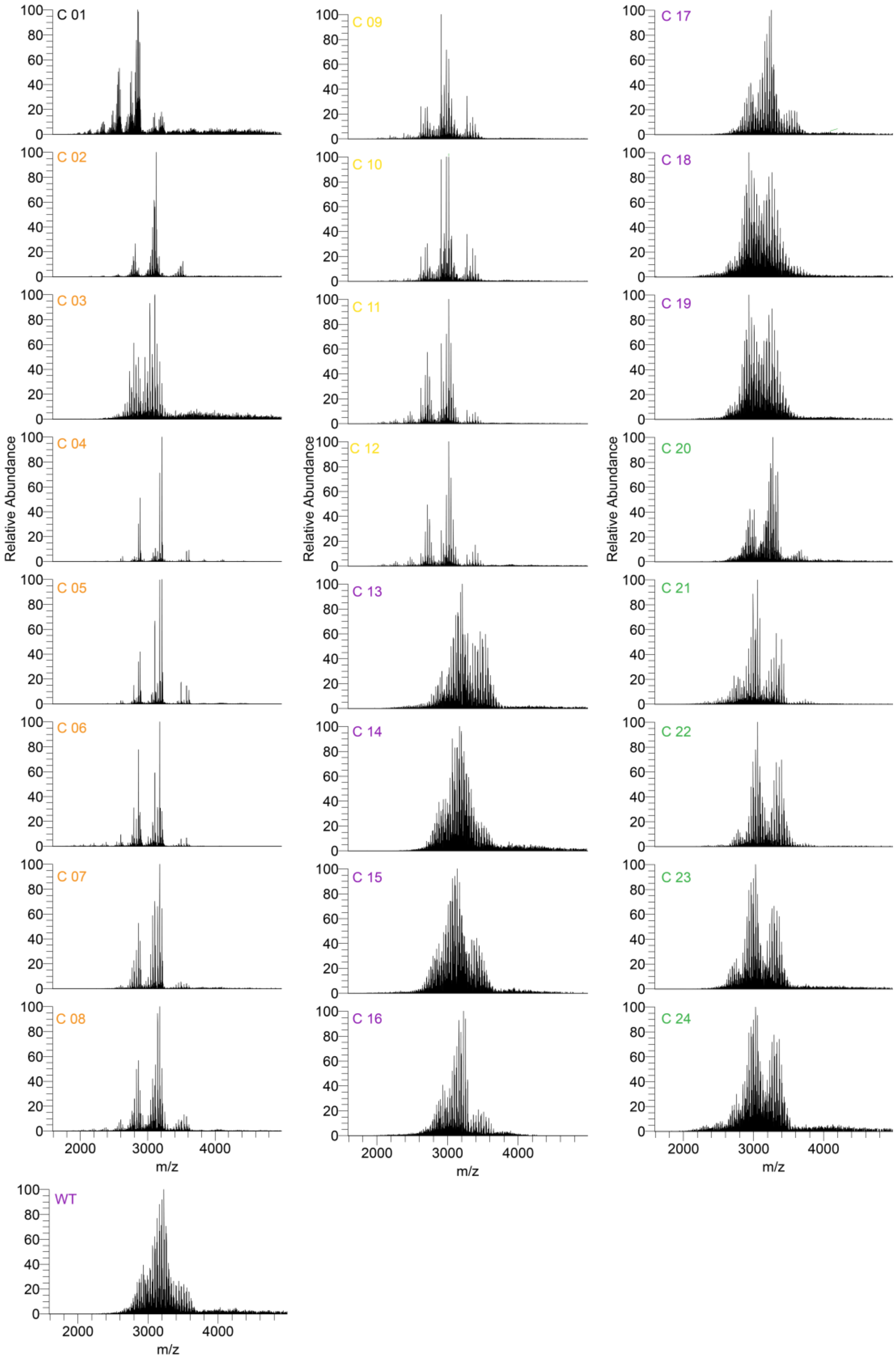

Supplementary Figure 4. Full raw native mass spectra of clones used in Figure 2. Clone names are shown in the inset of each spectrum and color coded according to the clusters depicted in **Figure 2.**

Supplementary Table 1. List of studied EPO clones. Enzyme knock-outs are described, and color coded according to the clusters observed in **Figure 2**.

EPO CLONE	Gene engineering	Effect
C 01	*mgat1* KO	High mannose glycans
C 02	*mgat3/4A/4B/5 cosmc* KO	Biantennary glycans + Tn antigen
C 03	*mgat2* KO	Loss of β2-Branch
C 04	KI *st6gal1* in *mgat4A/4B/5 st3gal4/6 -/-*	Homogenous biantennary α2,6-NeuAc sialylated glycans
C 05	KI *st6gal1* in *mgat4A/4B/5 st3gal4/6 -/-*	Biological replicate
C 06	*mgat4A/4B/5 st3gal4/6 B3gnt2* KO + *st6Gal-I* KI	Homogenous biantennary α2,6-NeuAc sialylated glycans
C 07	*B3gnt2/mgat4A/4B/5* KO	Biantennary glycans with eliminated polyLacNAc
C 08	*mgat4A/4B/5* KO	Biantennary sialylated glycans
C 09	*st3gal6* KO in *mgat4a/4b/5/ST3Gal4 -/-*	Biantennary glycans with decreased sialylation
C 10	*st3gal6* KO in *mgat4A/4B/5/st3gal4 -/-*	Biological replicate
C 11	*B3gnt2* KO in *mgat4A/4B/5 st3gal4/6 -/-*	Biantennary glycans with decreased sialylation and no polyLacNAc
C 12	*B3gnt2* KO in *mgat4A/4B/5 st3galt4/6 -/-*	Biological replicate
C 13	*fut8* KO	No fucosylation
C 14	*B4galt3* KO	No effect
C 15	*B3gnt1* KO	No effect
C 16	*B3gnt2* KO	Eliminated polyLacNAc
WT	/	Wild type
C 17	*B4galt4* KO	No effect
C 18	*st3gal4/6* KO	Decreased sialylation on N-glycans
C 19	*st3gal4/6* KO	Biological replicate
C 20	*mgat3/4A/5 cosmc* KO	Eliminates β6-branch of N-glycans, O-glycans all in the form of Tn
C 21	*mgat 5* KO	Eliminates β6-branch of N-glycans
C 22	*mgat4A/4B/5/st3gal4/6 cosmc* KO	UNKNOWN (sample mislabeled), native data indicate mgat4b
C 23	*mgat4A/4B* KO	Eliminates β4-branch of N-glycans
C 24	*mgat4B* KO	Eliminates β4-branch of N-glycans

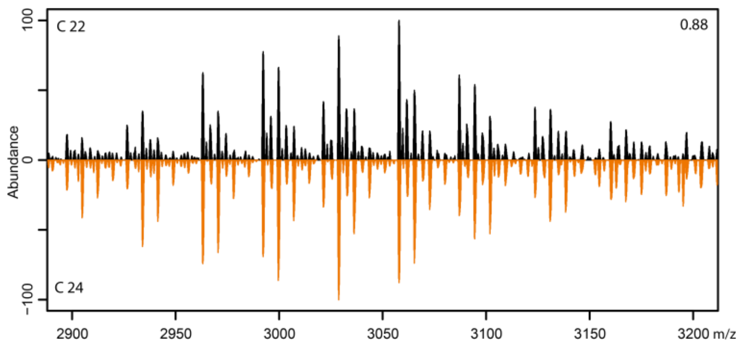

Supplementary Figure 5. Native mass spectra of mislabeled clone**.** Shown is the most abundant charge state (10+) of the mislabeled EPO C22 clone and for comparison that of EPO extracted from the clone C24 (orange). These data indicate that both clones share very similar glycoproteoform signatures as expressed by the high similarity score of 0.88 (Score is based on the full raw spectrums shown in **Supplementary Fig. 4)**

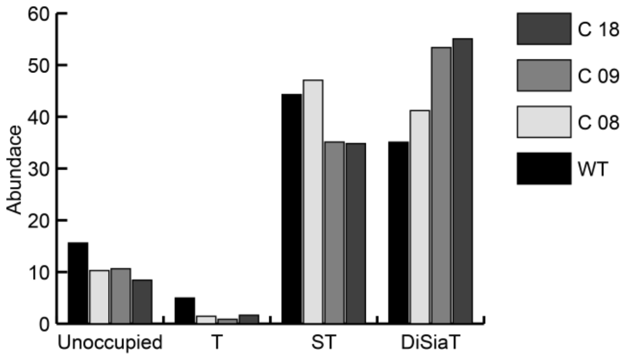

Supplementary Figure 6. Comparison of O-glycosylation upon *st3gal4/6* KO. WT and C 08 express tetra- and bi-antennary N-glycans, respectively, with normal sialylation, while C 18 and C 09 have an additional KO of *st3gal4/6* when compared to WT and C 08, respectively.

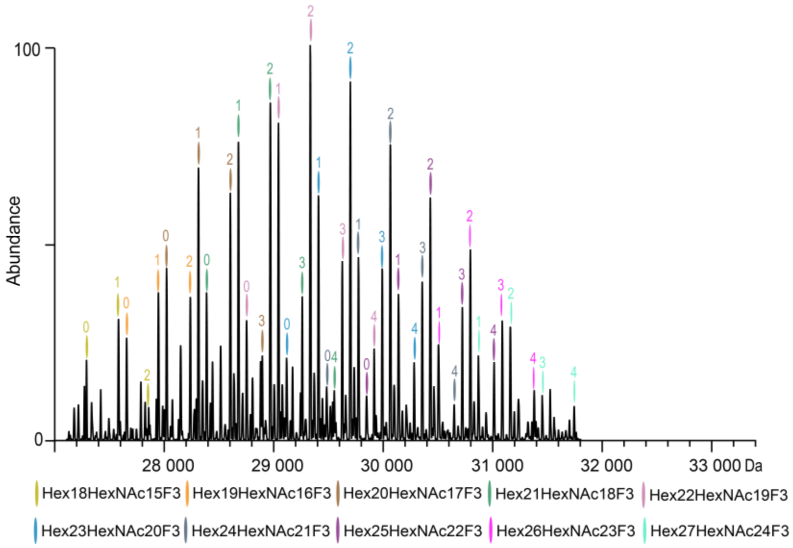

Supplementary Figure 7. Characterization of *st3gal4/6* KO influence on N-glycan sialylation. Main glycoproteoforms are color coded, wherein each color corresponds to a unique $Hex_{x+3}HexNAc_xF_3$ composition, and the numbers above the annotated peaks indicate the number of sialic acid residues.
